# Supplementary material for: Identification of photoperiod-induced specific miRNAs in the adrenal glands of Sunite sheep (Ovis aries)
Source: Front Vet Sci. 2022 Jul 22;9:888207. doi: 10.3389/fvets.2022.888207 (PMC9354845; doi:10.3389/fvets.2022.888207)
Supplement: Supplementary file 1 [file Data_Sheet_1.ZIP › Supplementary file/miRDeep2 file.zip!/miRDeep2 file/Know_Structure/SPLP42A2/oar-mir-29b-2.pdf]

miRBase precursor : oar-mir-29b-2
Total read count : 12485
oar-miR-29b read count : 11694
remaining reads : 791

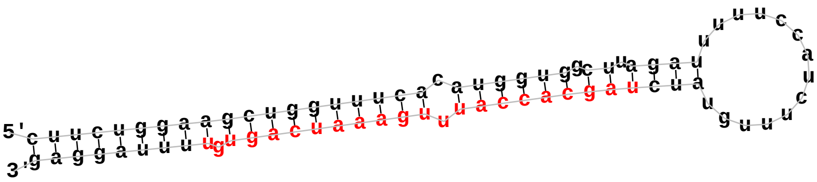

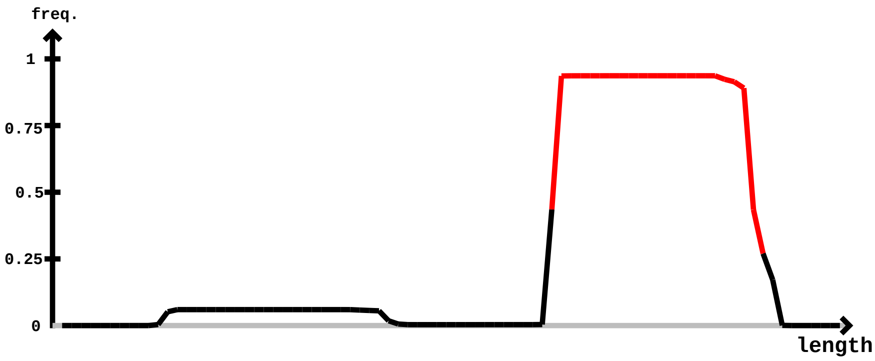

oar-miR-29b

```
5'- cuucuggaagcugguuucacaugguggcuuagauuuuuccaucuuuguaucuagcaccauuugaaaucaguguuuuaggag   -3'    exp
    (((((((((((((((((((.((((((.((.((((..............)))))))))))).)))))))))).)))))))))    reads      mm         sample
    .........gcugguuucacaugguggcu....................................................    1          0          seq
    .........gcugguuucacaugguggcuu...................................................    6          0          seq
    .........gcugguuucacaugguggcuua..................................................    7          0          seq
    .........gcugguuucacaugguggcuuag.................................................    3          0          seq
    .........gcugguuucacaugguggcuuaga................................................    31         0          seq
    .........gcugguuucacaugguggcuuagauu..............................................    1          0          seq
    ..........cugguuucacaugguggc.....................................................    1          0          seq
    ..........cugguuucacaugguggcuu...................................................    13         0          seq
    ..........cugguuucacaugguggcuua..................................................    11         0          seq
    ..........cugguuucacaugguggcuuag.................................................    9          0          seq
    ..........cugguuucacaugguggcuuaga................................................    424        0          seq
    ..........cugguuucacaugguggcuuagau...............................................    120        0          seq
    ..........cugguuucacaugguggcuuagauu..............................................    18         0          seq
    ...........ugguuucacaugguggcuu...................................................    1          0          seq
    ...........ugguuucacaugguggcuuag.................................................    4          0          seq
    ...........ugguuucacaugguggcuuaga................................................    55         0          seq
    ...........ugguuucacaugguggcuuagau...............................................    31         0          seq
    ...........ugguuucacaugguggcuuagauu..............................................    9          0          seq
    ...........ugguuucacaugguggcuuagauuu.............................................    1          0          seq
    ...............uucacaugguggcuuaga................................................    1          0          seq
    .................................uuGuuccaucuuuguau...............................    1          1          seq
    .................................uuuuuccaucuuuguauU..............................    7          1          seq
    .................................Cuuuuccaucuuuguauc..............................    1          1          seq
    .................................uuuuuccaucuuuguauc..............................    34         0          seq
    ...........................................uuuguaucuagcaccauuugaaaucaguguu.......    1          0          seq
    .................................................ucuagcaccauuugaaaucagu..........    6          0          seq
    ..................................................cuagcaccauuugaaau..............    1          0          seq
    ..................................................cuagcUccauuugaaauc.............    1          1          seq
    ..................................................cuagcaccauuugaaauc.............    58         0          seq
    ..................................................cuaCcaccauuugaaauc.............    1          1          seq
    ..................................................cuagcaccauuugaaaucG............    5          1          seq
    ..................................................cuagcaccauuugaaauca............    31         0          seq
    ..................................................cuagcaccauuugaaaucGg...........    3          1          seq
    ..................................................cuagcaccauuugaaaucag...........    31         0          seq
    ..................................................cuagcaccauuugaaaucagu..........    2923       0          seq
```

oar-miR-29b

```
cuucuggaagcugguuucacaugguggcuuagauuuuuccaucuuuguaucuagcaccauuugaaaucaguguuuuaggag
..................................................cuagcaccauuugaaaucGgu..........    31      1       seq
..................................................cuagcaccauuugaaaucagug.........    1508    0       seq
..................................................cuagcaccauuugaaaucagugu........    794     0       seq
..................................................cuagcaccauuugaaaucaguguu.......    3       0       seq
...................................................uagcaccauuugaaauU.............    4       1       seq
...................................................uagcaccauuugaaauA.............    1       1       seq
...................................................uagcaccauuugaaauc.............    102     0       seq
...................................................uagcaccauuugaaaucG............    44      1       seq
...................................................uagcaccauuugaaauca............    34      0       seq
...................................................uagcaccauuugaaaucag...........    58      0       seq
...................................................uagcaccauuugaaaucGg...........    201     1       seq
...................................................uagcacAauuugaaaucag...........    1       1       seq
...................................................uagcaccauuugaaaucGgu..........    698     1       seq
...................................................uagcaccauuugaaaucagu..........    2033    0       seq
...................................................uagcaccauuugaaaucagug.........    547     0       seq
...................................................uagcaccauuugaaaucagugu........    432     0       seq
...................................................uagcaccauuugaaaucaguguu.......    2125    0       seq
...................................................uagcaccauuugaaaucaguguuC......    1       1       seq
...................................................uagcaccauuugaaaucaguguuu......    8       0       seq
....................................................agcaccauuugaaaucagu..........    1       0       seq
....................................................agcaccauuugaaaucaguguu.......    5       0       seq
.....................................................gcaAcauuugaaaucag...........    1       1       seq
.....................................................gcaccauuugaaaucaguguu.......    1       0       seq
........................................................ccauuugaaaucaguguG.......    1       1       seq
```
